# Correction: Comparative pharmacokinetics of four active components on normal and diabetic rats after oral administration of Gandi capsules

**DOI:** 10.1039/c8ra90016g

**Published:** 2018-02-26

**Authors:** Renjie Xu, Jia Qi, Ruan-Juan Zhan, Gui-Sheng Zhou, Bin Hao, Jing Ma, Xin Wei, AJing Xu, Jian Zhang

**Affiliations:** Department of Pharmacy, Xinhua Hospital, Shanghai Jiaotong University School of Medicine No. 1665 Kongjiang Road, Yangpu District Shanghai 200092 China zhangjian@xinhuamed.com.cn xuajing@xinhuamed.com.cn +86-21-25077150 +86-21-25077150 +86-21-25077156; Department of Pharmacy, The First Affiliated Hospital, Wenzhou Medical University Wenzhou China; Jiangsu Collaborative Innovation Center of Chinese Medicinal Resources Industrialization, National and Local Collaborative Engineering Center of Chinese Medicinal Resources Industrialization and Formulae Innovative Medicine, Nanjing University of Chinese Medicine No. 138 Xianlin Road Nanjing 210023 China; School of Pharmacy, Shanghai Jiao Tong University No. 800 Dongchuan Road, Minhang District Shanghai 200240 China

## Abstract

Correction for ‘Comparative pharmacokinetics of four active components on normal and diabetic rats after oral administration of Gandi capsules’ by Renjie Xu *et al.*, *RSC Adv.*, 2018, **8**, 6620–6628.

AJing Xu's name, affiliation and E-mail address were incorrectly reproduced in the published article. The correct versions are shown here.

The Royal Society of Chemistry apologises for these errors and any consequent inconvenience to authors and readers.

## Supplementary Material

